# Conflict-Specific Aging Effects Mainly Manifest in Early Information Processing Stages—An ERP Study with Different Conflict Types

**DOI:** 10.3389/fnagi.2016.00053

**Published:** 2016-03-16

**Authors:** Margarethe Korsch, Sascha Frühholz, Manfred Herrmann

**Affiliations:** ^1^Department of Neuropsychology and Behavioral Neurobiology, Bremen UniversityHochschulring, Bremen, Germany; ^2^Center for Cognitive Sciences (ZKW), Bremen UniversityHochschulring, Bremen, Germany; ^3^Department of Psychology, University of ZurichZurich, Switzerland

**Keywords:** P2, N2, Fanker conflict, stimulus-response conflict, event-related potentials, aging

## Abstract

Aging is usually accompanied by alterations of cognitive control functions such as conflict processing. Recent research suggests that aging effects on cognitive control seem to vary with degree and source of conflict, and conflict specific aging effects on performance measures as well as neural activation patterns have been shown. However, there is sparse information whether and how aging affects different stages of conflict processing as indicated by event related potentials (ERPs) such as the P2, N2 and P3 components. In the present study, 19 young and 23 elderly adults performed a combined Flanker conflict and stimulus-response-conflict (SRC) task. Analysis of the reaction times (RTs) revealed an increased SRC related conflict effect in elderly. ERP analysis furthermore demonstrated an age-related increase of the P2 amplitude in response to the SRC task. In addition, elderly adults exhibited an increased P3 amplitude modulation induced by incongruent SRC and Flanker conflict trials.

## Introduction

With increasing age several alterations of the brain such as changes of brain structure, different transmitter systems and the accumulation of pathological processes become evident (see Park and Reuter-Lorenz, 2009). According to the frontal lobe hypothesis (West, 1996) these changes especially manifest in the prefrontal cortex. As a consequence, cognitive functions that are linked to the prefrontal cortex such as interference control are highly susceptible to aging processes. Interference control refers to the ability to inhibit the processing of irrelevant information and incorrect reactions. Indeed, different studies show that elderly individuals are slower and commit more errors in tasks that require inhibitory functions (van der Lubbe and Verleger, 2002; Kubo-Kawai and Kawai, 2010). To investigate interference control experimental conflict designs such as the Flanker conflict (Eriksen and Eriksen, 1974) or Simon conflict (Simon, 1969) tasks are employed. The Flanker task typically consists of a central target stimulus that is surrounded by irrelevant Flanker stimuli either congruent or incongruent with the target stimulus. While congruent Flanker stimuli usually facilitate fast and correct responses, trials with incongruent stimulus input are associated with increased reaction times (RTs) and higher error rates. This task is considered to represent a stimulus-stimulus interference task (S-S; according to the taxonomy of Kornblum et al., 1990) and will be further referred to as Flanker interference condition. The Simon task is characterized by irrelevant spatial information induced for example by a lateralized presentation of a target stimulus. When response and presentation side correspond (congruent condition) responses are faster and fewer errors are committed than in trials with non-corresponding presentation and response side (incongruent condition). In contrast to the Flanker task, the Simon conflict is considered a stimulus-response conflict (further referred to as stimulus-response-conflict (SRC) task; see Kornblum et al., 1990). Several studies indicate that the neural correlates of the different conflict types seem to be dissociated with regard to their spatial and temporal characteristics. Thus, interference control seems to consist of different subcomponents and does not represent a unitary function. Importantly, there is increasing evidence that aging does not affect all subcomponents and -processes equally but rather shows conflict specific modifications of interference control (Kawai et al., 2012; Sebastian et al., 2013). The specificity of aging effects on conflict processing is reflected by differential effects on behavioral measures: increased conflict effects with regard to RT and error rates in elderly individuals are frequently detected in SRC tasks (van der Lubbe and Verleger, 2002; Kubo-Kawai and Kawai, 2010), while in many experiments introducing the Flanker task no significant deterioration of task performance is found in elderly (Falkenstein et al., 2001; Nieuwenhuis et al., 2002; Fernandez-Duque and Black, 2006; Hsieh and Fang, 2012). These differences can also be demonstrated when both conflicts are investigated in the same group (Kawai et al., 2012; Korsch et al., 2014).

Moreover, several fMRI studies reported task specific aging effects on the activation patterns associated with different types of executive and inhibitory functions (Kawai et al., 2012; Turner and Spreng, 2012; Sebastian et al., 2013). In a recent fMRI study (Korsch et al., 2014) we furthermore demonstrated differential effects of aging on the neural substrates of SRC and Flanker conflict processing: we found that elderly adults exhibited extended activation when compared to their younger peers in response to the Flanker conflict, however, both groups recruited similar regions such as caudate nucleus, frontal and occipital regions. These data indicate that both groups resolve the Flanker conflict in a similar fashion. In contrast, the SRC task elicited different activation patterns in both groups: while elderly individuals showed activation in frontal and parietal areas, in younger adults mainly deactivation became evident. These markedly different activation patterns suggest that there is a qualitative age-related difference in the processing of the SRC.

However, there are only few reports on the specificity of aging effects on the temporal dynamics of conflict processing. There is considerable evidence from event-related potential (ERP) studies that early as well as later processing stages of interference control are differentially modified by distinct conflict types in young adults (Frühholz et al., 2011; Wang et al., 2014). In the present study, we therefore decided to investigate different time windows of conflict processing using ERPs that are known to be involved in conflict resolution such as the N2 and P3. The N2 is an early component characterized by a negative deflection occurring 250–300 ms after stimulus onset. It can be subdivided to different subcomponents depending on the topography and the underlying cognitive processes (for reviews about N2, see Folstein and Van Petten, 2008; Larson et al., 2014). Conflict tasks and other tasks that require response inhibition modulate the N2 subcomponent that appears over frontal electrode sites. The amplitude of this anterior N2 is enhanced in conditions with an increased demand for inhibition such as incongruent conflict task trials. These findings led to the assumption that N2 seems to be associated with control mechanisms like response inhibition and conflict monitoring (Yeung et al., 2004; Folstein and Van Petten, 2008). This line of evidence was furthermore substantiated by source analysis studies that demonstrated neural generators of the N2 in the medial wall of the frontal cortex (Bekker et al., 2005; Wascher et al., 2011), a region that is frequently activated during monitoring tasks and response selection. Another early component that might be modified by inhibitory control processing is the P2 component. The P2 component appears after 150–250 ms after stimulus onset over frontal electrode sites. Gajewski et al. (2008) reported that the P2 amplitude is increased in incompatible stimulus conditions. West et al. (2004) additionally found that in a numerical conflict task stimulus incongruence also lead to an increase of the P2 amplitude. The amplitude enhancement in trials with incongruent stimuli might thus reflect an increased effort of stimulus evaluation (Potts, 2004; Gajewski et al., 2008).

Both ERP components were found to change with increasing age. A delay of N2 latency (Falkenstein et al., 2002) and a significant decrease of amplitude of the N2 in elderly individuals have been reported across different tasks (Czigler et al., 1996; Bertoli and Probst, 2005; Wascher et al., 2011; Lucci et al., 2013). In accordance with these findings, Wild-Wall et al. (2008) also reported that elderly exhibited a reduced N2 amplitude in a Flanker task which was attributed to a reduction of perceived response conflict due to a change of processing strategy. Whether Flanker conflict and SRC processing show different modulations of the N2 component or whether aging shows a conflict specific impact on the N2 is still unclear. There is evidence that aging does not generally affect the N2 component but rather shows specific effects for different processing contexts (Falkenstein et al., 2002). There is sparse literature on how aging might affect the P2 component in conflict processing. However, age-related changes of the P2 component can be observed in studies investigating working memory and novelty processing (McEvoy et al., 2001; Daffner et al., 2015). Furthermore, the age-related enhancement of the P2 in a Stroop task (West and Alain, 2000; Zurron et al., 2014) clearly demonstrates that the P2 component seems significantly associated with aging effects on interference control processing.

The later P3 component is also reported to be modified by different conflict tasks. Comparable with the N2, the P3 can be subdivided in different subcomponents with distinct topographies, and reflecting different cognitive processes. The parietal P3b component seems to be related to the allocation of attentional resources (Polich and Heine, 1996) and might additionally indicate the timing of stimulus evaluation (Kutas et al., 1977; Duncan-Johnson and Kopell, 1981). The P3b amplitude and latency are modulated by both the Flanker and the Simon task (Van’t Ent, 2002; Umebayashi and Okita, 2010; Frühholz et al., 2011). The effects of aging on the P3 component are characterized by a decrease of amplitude and a delay of latency (Dujardin et al., 1993; Polich, 1996). This effect was also illustrated in the context of conflict processing (van der Lubbe and Verleger, 2002; Wild-Wall et al., 2008). In contrast to the N2 Falkenstein et al. (2002) reported the P3 component to be equally affected by aging in different stimulation contexts. The authors argued that cognitive processes indexed by the P3 might be less specific to different components of inhibition when compared to the N2 component.

The aim of the present study was to investigate whether aging affects specific stages of conflict processing in different conflict task conditions. Therefore, elderly adults and younger controls underwent EEG recording with a combined SRC and Flanker conflict task. Based on previous data, we hypothesized that aging differentially affects the respective stages of conflict processing and that in particular the processing of the SRC task will be altered in elderly individuals. Thus, we expect that behavioral SRC effects as indicated by RT and/or error rates will be increased in elderly individuals. Both, early and late phases of information processing are modulated by interference and aging processes. However, the age-related modulation of early phases seems to be more specific to different contexts of interference (Falkenstein et al., 2002; Frühholz et al., 2011). We thus hypothesized that conflict specific effects will particularly manifest in the P2 or the N2 time window.

## Materials and Methods

### Participants

Twenty young and 25 elderly subjects participated in the present study. One male participant in each group had to be excluded from further analysis due to massive movement artifacts in EEG data. Additionally, a comprehensive neuropsychological examination revealed minor cognitive deficits in one male elderly participant, who was also excluded from further analysis. Thus, the final study sample consists of 19 young (10 male, mean age 23.05 years, SD = 2.76) and 23 elderly adults (11 male, mean age 70.32 years, SD = 3.24). All participants were right-handed according to the Edinburgh Handedness Inventory (Oldfield, 1971), had normal or corrected to normal vision, and reported no prior history of psychiatric or neurological disorders. All participants gave informed and written consent for participation. The study was approved by the local ethics committee of the University of Bremen.

### Experimental Tasks

**Figure 1:**
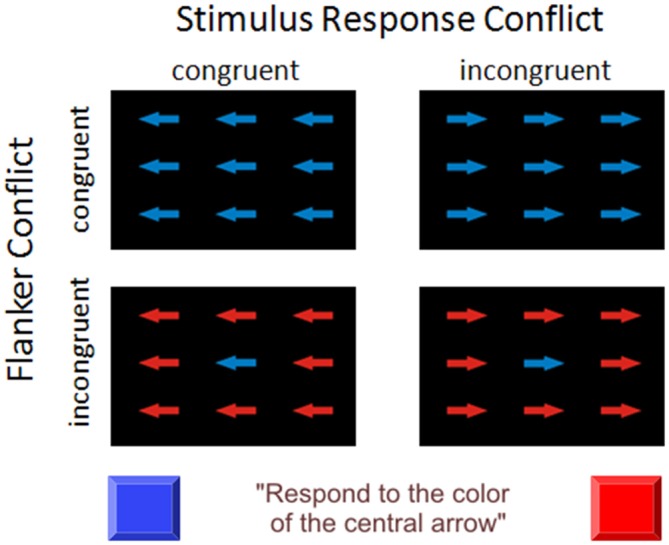
**Experimental task design.** The participants were instructed to attend to the color of the central arrow (blue or red) and to react as fast as possible via right or left button press with their index finger. The arrows surrounding the central target could either have the same color as the target (congruent Flanker condition) or the color allocated to the opposite response side (incongruent Flanker condition). Furthermore, all arrows were either oriented to the correct response side as indicated by the color of the central arrow (congruent stimulus-response-conflict (SRC) condition) or pointed to the opposite direction (incongruent SRC condition). There were four experimental conditions: (1) congruent Flanker and SR condition (FcSc); (2) incongruent Flanker and congruent SRC condition (FiSc); (3) congruent Flanker and incongruent SRC condition (FcSi), and (4) incongruent Flanker and SRC condition (FiSi). In the depicted scenario the color “blue” of the central arrow is associated with the left response side and the color “red” with the right response side. The association of color and response side was counterbalanced across participants.

We introduced a Flanker conflict task and a Simon-like (SRC) task condition as well as a combination of both conflict types (see Figure 1). The stimulus material was derived from previous studies (Frühholz et al., 2011; Korsch et al., 2014) and consisted of nine arrows arranged in three rows. Participants were instructed to attend to the color of the central arrow (blue or red) and to react as fast as possible via right or left button press with their index finger. The arrows surrounding the central target could either have the same color as the target (congruent Flanker condition) or the color allocated to the opposite response side (incongruent Flanker condition). Furthermore, all arrows were either oriented to the correct response side as indicated by the color of the central arrow (congruent SRC condition) or pointed to the opposite direction (incongruent SRC condition). Thus, there were four experimental conditions: (1) congruent Flanker and SRC condition (FcSc); (2) incongruent Flanker and congruent SRC condition (FiSc); (3) congruent Flanker and incongruent SRC condition (FcSi); and (4) incongruent Flanker and SRC condition (FiSi). Stimuli were presented in six blocks of 60 trials separated by short breaks of 10 s. There were 72 trials of each condition. Trial sequence was pseudo-randomized to avoid effects of trial succession. Each trial started with a white fixation cross on black background in the center of the screen (800 ± 150 ms). Thereafter, the set of arrows appeared for 250 ms followed by a blank screen for 2000 ms. Stimuli were presented on a computer screen (refresh rate 60 Hz) using Presentation Software (neurobehavioral Systems; https://www.neurobs.com/menu_presentation/menu_features/features_overview). Participants were seated in front of the screen with a viewing distance of 60 cm. The experiment was conducted in a darkened room. Before or after the present experiment the participants completed an fMRI session with the same task (Korsch et al., 2014).

### EEG Recording

EEG signal was recorded using 64 Ag-AgCl electrodes placed on an elastic cap according to the 10–10-system with a sampling rate of 512 Hz. Interconnected ear lobe electrodes were used as reference. EEG signal was amplified by a REFA^®^ multi-channel system (TMS International; www.tmsi.com), and impedance threshold was set to 10 kΩ. Eye movements and blinks were monitored by vertical and horizontal electrooculograms. Offline data processing was performed with the Brain Electrical Source Analysis Software (BESA^®^, Version 5.1.8.10, MEGIS Software GmbH, Gräfelfing, Germany), and comprised band-pass filtering (0.1–20 Hz) and rereferencing to average reference. Trials were averaged stimulus-locked within a time frame of 100 ms before and 800 ms after stimulus onset. EOG artifacts were corrected using the algorithm introduced by Ille et al. (2002). Each trial was visually inspected, and incorrect trials and trials with artifact contamination were discarded from further analysis. To avoid differences of trial number between conditions and groups 50 (±10) trials equally distributed over the six runs were included in the EEG data analysis.

### Statistical Analysis

**Figure 2:**
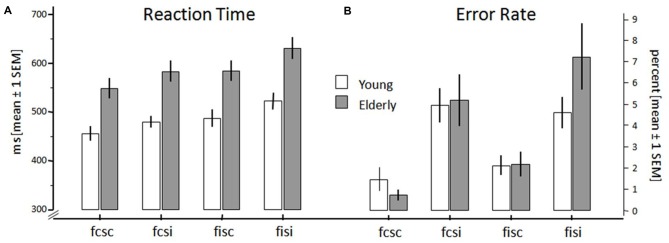
**Reaction times (RTs; A) and error rates (B) of young and elderly participants for the conditions fcsc (both Flanker and SRC congruent), fisc (Flanker incongruent, SRC congruent), fcsi (Flanker congruent, SRC incongruent), and fisi (both Flanker and SRC incongruent).** Error bars show standard error of mean.

According to our hypotheses we focused data analyses on the P2, N2, and P3. The respective time windows for the P2 (140–220 ms), N2 (240–330 ms) and P3 (310–570 ms) components were determined by visual inspection of the grand average waveforms of both groups. Due to different scalp distributions of the P2, N2 and P3 components, we included different electrode positions in the analyses of the respective components. EEG data show P2 and N2 to be particularly marked over frontal and frontocentral electrode positions in both groups (see Figure 2). Thus, for the analyses of the P2 and N2 components peak amplitudes were extracted for the electrodes F1, Fz, F2, FC1, FCz and FC2 for each participant in the respective time frames. In contrast to the P2 and the N2, the P3 was characterized by a plateau-like shape, and we thus decided to calculate mean amplitude for both groups. Since the P3 had different onsets in both age groups, the different time frames were determined for the respective groups (young: 310–430 ms; elderly: 450–570 ms). The P3 was most pronounced over parietal and centroparietal recording sites in both groups, and we thus decided to include the P1, Pz, P2, CP1, CPz and CP2 electrodes in the analysis. Amplitudes were entered into a 3 × 2 × 2 × 2 × 2 analysis of variance (ANOVA) with *Laterality* of electrode position (right vs. midline. vs. left electrodes), Frontality (P2 and N2: frontocentral vs. frontal electrode; P3: parietal vs centroparietal electrodes), *Flanker* (congruent vs. incongruent) and *SRC* (congruent vs. incongruent) as within-subject factors, and *Age* (young vs. elderly) as between-subject factor. *Post hoc* analyses were based on *t*-tests with a significance level of α = 0.05 (Bonferroni corrected for multiple comparisons). The Greenhouse-Geisser correction was applied for violations of sphericity.

## Results

### Behavioral Data

In order to compensate for extreme values and outliers in the behavioral raw data we calculated the median reaction times of all subjects. Thereafter, these data together with the error rates of participants were each entered into a 2 × 2 × 2 ANOVA with the within-subject factors *Flanker* (congruent vs. incongruent) and *SRC* (congruent vs. incongruent), and the between-subject factor *Age* (young vs. elderly). Figure 2 shows RTs and error rates for both groups and all conflict conditions. The RT analysis (Figure 2A) revealed a significant main effect for the factors *Flanker* (*F*_(1,40)_ = 146.60, *p* < 0.001) and *SRC* (*F*_(1,40)_ = 77.51, *p* < 0.001). Both conflict types elicited longer RT in incongruent (Flanker: M = 536 ms; SEM = 11.88; SRC: M = 534 ms; SEM = 12.62) compared to congruent trials (Flanker: M = 494 ms; SEM = 12.51; SRC: M = 497 ms; SEM = 11.89). In addition, the interaction of SRC × Flanker reached significance (*F*_(1,40)_ = 21.22, *p* < 0.001). The RT difference of congruent and incongruent trials was larger in trials with two sources of incongruent information. The Flanker congruency effect was larger in SRC incongruent (Δ: M = 51 ms; SEM = 3.79) than in SRC congruent trials (Δ: M = 33 ms; SEM = 4.06; *p* < 0.001). Additionally, the SRC congruency effect was larger in trials with incongruent Flanker (Δ: M = 47 ms; SEM = 5.00) stimuli than in trials with congruent Flanker stimuli (Δ: M = 28 ms; SEM = 4.51; *p* < 0.001). The factor *Age* also showed significant differences (*F*_(1,40)_ = 18.60, *p* < 0.001). RTs were increased in elderly (M = 567 ms; SEM = 16.25) compared to young adults (M = 463 ms; SEM = 17.88). Additionally, there was a significant interaction of *Age* × *SRC* (*F*_(1,40)_ = 4.50, *p* = 0.040). The SRC congruency effect (incongruent-congruent) was significantly larger in elderly (M = 46 ms; SEM = 4.52) than in young (M = 28 ms; SEM = 6.59) participants (*p* = 0.040).

There was also a significant main effect for the factor SRC (*F*_(1,40)_ = 28.71, *p* < 0.001) regarding the error rates (Figure 2B). All participants committed more errors in incongruent SRC (M = 5.50%; SEM = 0.78) trials when compared to the congruent SRC condition (M = 1.62%; SEM = 0.24). However, there was no significant effect for the factors *Age* (*F*_(1,40)_ = 0.38, *p* = 0.540) and *Flanker* (*F*_(1,40)_ = 3.87, *p* = 0.056).

### Electrophysiological Data

**Figure 3:**
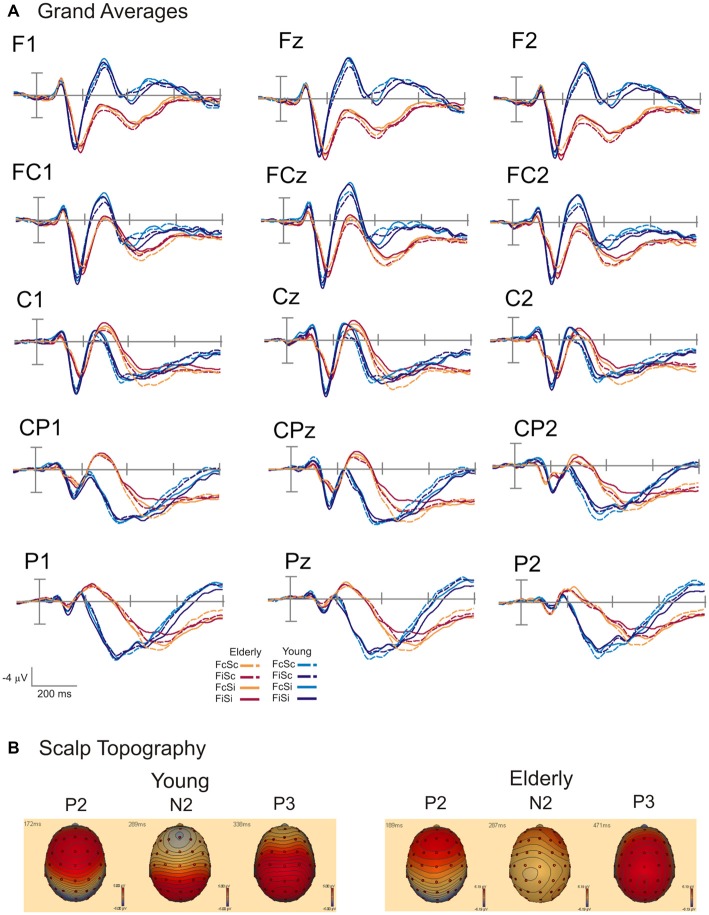
**(A)** Group-averaged event-related potentials (ERPs) for the conditions FcSc (Flanker and SRC congruent), FiSc (Flanker incongruent, SRC congruent), FcSi (Flanker congruent, SRC incongruent) and FiSi (Flanker and SRC incongruent) for young and elderly adults. Stimulus onset is shown by the vertical line. Ticks on the time axis represent 200 ms. **(B)** Topography of the P2, N2, and P3 component for young and elderly participants.

**Figure 4:**
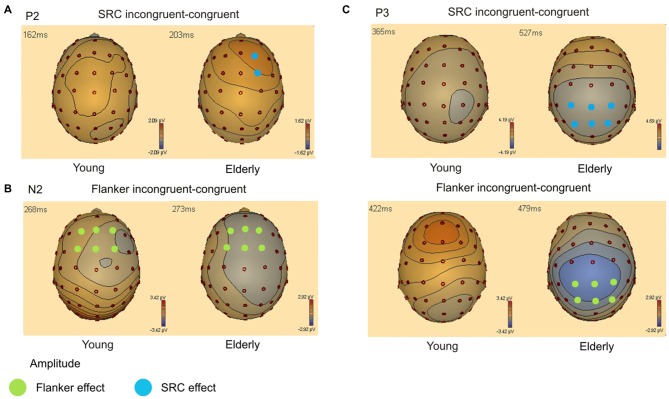
**(A)** Topography of amplitude difference between SRC incongruent—congruent condition for the P2 component (140–220 ms). Blue circles indicate electrodes with a significant SRC amplitude effect. **(B)** Topography of amplitude difference between Flanker incongruent—congruent condition for the N2 component (240–330 ms). Green circles represent electrodes with a significant Flanker conflict amplitude effect. **(C)** Topography of amplitude difference between SRC and Flanker incongruent—congruent condition for the P3 component (310–570 ms). Colored circles indicate significant SRC (blue) and Flanker conflict (green) amplitude effects.

Grand averages and scalp topography of P2, N2 and P3 are shown in Figure 3. Figure 3A displays the grand average waveforms for young and elderly adults over parietal, central and frontal electrodes. Figure 3B illustrates the scalp topography for three time points referring to the P2, N2, and P3 components. The topography of the P2 component is characterized by a peak amplitude over frontal and frontocentral electrodes in both groups. The N2 also shows a frontal distribution in young individuals. In elderly the N2 is located more posteriorly. The P3 demonstrates a centroparietal orientation in young and elderly participants. The effects of the respective conflict tasks in both groups are displayed in Figure 4. Furthermore, results of the ANOVAs for the different components are presented in Tables 1, 2.

**Table 1:** **Overview of the mean values (μV) and standard errors (SE) of the P2 and N2 amplitude analysis**.

P2		Mean (μV; SE)
**Flanker**
Con		5.25 (0.35)
Incon		5.74 (0.35)
**Age × Frontality**
Eldery	F	5.58 (0.50)
	FC	5.23 (0.46)
Young	F	5.37 (0.55)
	FC	5.80 (0.51)
**Age × SRC × Laterality**
Elderly
Right	Con	5.21 (0.48)
	Incon	5.88 (0.57)
Midline	Con	5.38 (0.53)
	Incon	5.63 (0.56)
Left	Con	5.12 (0.55)
	Incon	5.32 (0.54)
Young
Right	Con	4.93 (0.46)
	Incon	5.00 (0.42)
Midline	Con	5.73 (0.50)
	Incon	5.75 (0.45)
Left	Con	5.41 (0.41)
	Incon	5.53 (0.41)

**N2**		**Mean (μV; SE)**

**Age**
Elderly		0.17 (0.52)
Young		−3.40 (0.58)
**Frontality**
Frontal		−1.40 (0.43)
Frontocentral		−1.83 (0.38)
**Laterality**
Right		−1.26 (0.38)
Midline		−1.93 (0.42)
Left		−1.65 (0.39)
**Age × Frontality**
Eldery	F	0.86 (0.58)
	FC	−0.51 (0.51)
Young	F	−3.65 (0.64)
	FC	−3.15 (0.56)
**Age × Laterality**
Eldery	Right	0.08 (0.57)
	Midline	0.60 (0.51)
	Left	−0.10 (0.53)
Young	Right	−3.87 (0.63)
	Midline	−3.12 (0.56)
	Left	−3.21 (0.58)
**Flanker**
Con		−1.34 (0.41)
Incon		−1.88 (0.38)
**Age × SRC × Laterality**
Elderly
Right	Con	0.71 (0.50)
	Incon	0.50 (0.46)
Midline	Con	−0.10 (0.50)
	Incon	0.12 (0.54)
Left	Con	−0.11 (0.50)
	Incon	−0.08 (0.48)
Young
Right	Con	−3.15 (0.59)
	Incon	−3.09 (0.62)
Midline	Con	−3.88 (0.72)
	Incon	−3.85 (0.66)
Left	Con	−3.29 (0.64)
	Incon	−3.13 (0.62)

*Values are arranged by significant main effects and interactions (factors written in bold letters) extracted from the respective ANOVAs. F: frontal; FC: frontocentral; con: congruent; incon: incongruent*.

**Table 2:** **Overview of the mean values (μV) and standard errors (SE) of the P3 amplitude analysis**.

P3		Mean (μV; SE)
**Flanker**
Con		4.04 (0.36)
Incon		3.48 (0.34)
**Laterality**
Right		3.43 (0.33)
Midline		4.02 (0.37)
Left		3.81 (0.37)
**SRC**
Con		4.05 (0.36)
Incon		3.47 (0.34)
**Age × SRC × Laterality**
Elderly
Right	Con	2.70 (0.49)
	Incon	3.38 (0.51)
Midline	Con	4.08 (0.60)
	Incon	3.22 (0.56)
Left	Con	3.78 (2.81)
	Incon	2.96 (0.54)
Young
Right	Con	3.56 (0.41)
	Incon	4.09 (0.43)
Midline	Con	4.56 (0.44)
	Incon	4.24 (0.433)
Left	Con	4.39 (0.45)
	Incon	4.13 (0.46)
**Age × Flanker/Age × Flanker ×**
**Frontality × Laterality**
Elderly
CP1	Con	3.75 (0.67)
	Incon	2.83 (0.62)
CPz	Con	3.17 (0.62)
	Incon	4.09 (0.62)
CP2	Con	2.73 (0.51)
	Incon	3.55 (0.56)
P1	Con	3.08 (0.50)
	Incon	3.81 (0.59)
	Con	4.01 (0.59)
	Incon	3.31 (0.52)
P2	Con	3.34 (0.53)
	Incon	2.56 (0.47)
Young
CP1	Con	4.11 (0.40)
	Incon	4.03 (0.44)
CPz	Con	4.13 (0.42)
	Incon	4.34 (0.38)
CP2	Con	3.42 (0.39)
	Incon	3.99 (0.30)
P1	Con	4.33 (0.52)
	Incon	4.56 (0.50)
Pz	Con	4.79 (0.52)
	Incon	4.34 (0.50)
P2	Con	4.11 (0.50)
	Incon	3.77 (0.55)

*Values are arranged by significant main effects and interactions (factors written in bold letters) extracted from the ANOVAs. CP1, CPz, CP2, P1, Pz, P2 represent electrode positions; con: congruent; incon: incongruent*.

#### P2 Time Window

Peak amplitude analysis revealed a significant effect of the factor *Flanker* (*F*_(1,40)_ = 27.75, *p* < 0.001). Incongruent Flanker trials (M = 5.74 μV; SEM = 0.35) elicited higher amplitudes compared to congruent Flanker trials (M = 5.25 μV; SEM = 0.35). The factors *Age* (*F*_(1,0)_ = 0.65, *p* = 0.800) and *SRC* (*F*_(1,0)_ = 2.38, *p* = 0.131) did not reach significance, however, there was a significant interaction of the factors *SRC* × *Age* × *Laterality* (*F*_(2,39)_ = 5.28, *p* = 0.007). This interaction resulted from a significant SRC congruency effect over right electrodes (F2 and FC2) in elderly (congruent: M = 5.21 μV; SEM = 0.48; incongruent: M = 5.88 μV; SEM = 0.57; *p* = 0.004) which was not detectable in young participants (congruent: M = 4.93 μV; SEM = 0.46; incongruent: M = 5.00 μV; SEM = 0.42; *p* = 0.674). Furthermore, there was a significant interaction of *Frontality* × *Age* indicating that the P2 amplitude was most pronounced over frontal electrodes in elderly individuals (frontal: M = 5.58 μV; SEM = 0.50; frontocentral: M = 5.23 μV; SEM = 0.46), while young adults exhibited enhanced amplitudes over frontocentral recording sites (frontal: M = 5.37 μV; SEM = 0.55; frontocentral: M = 5.80 μV; SEM = 0.51).

#### N2 Time Window

The analysis of the N2 component yielded a significant effect of the factor *Flanker* (*F*_(1,40)_ = 22.28, *p* < 0.001). Amplitudes were more negative in the incongruent Flanker condition (M = −1.88 μV; SEM = 0.38) than the congruent Flanker condition (M = −1.34 μV; SEM = 0.41). Moreover, there were significant effects for the factors *Laterality* (*F*_(2,39)_ = 14.62, *p* < 0.001), *Frontality* (*F*_(1,40)_ = 4.31, *p* = 0.044), and *Age* (*F*_(1,40)_ = 20.80, *p* < 0.001). There was also an interaction of *Laterality* × *Age* (*F*_(2,39)_ = 5.22, *p* = 0.007) and *Frontality* × *Age* (*F*_(1,40)_ = 19.91, *p* < 0.001). These interactions indicate that the N2 component was most prominent over frontocentral (frontal: M = 0.86 μV; SEM = 0.58; frontocentral: M = −0.51 μV; SEM = 0.51) and left-sided electrodes (left: M = −0.10 μV; SEM = 0.526; midline: M = 0.6 μV; SEM = 0.51; right: M = 0.08 μV; SEM = 0.57) in elderly. In contrast, the N2 peaked over frontal electrodes (frontal: M = −3.65 μV; SEM = 0.64; frontocentral: M = −3.15 μV; SEM = 0.56) and was most pronounced over midline electrodes (left: M = −3.21 μV; SEM = 0.58; midline: M = −3.12 μV; SEM = 0.56; right: M = −3.87 μV; SEM = 0.63) in younger adults. There was no significant effect for the factor SRC (*F*_(1,40)_ = 1.96, *p* = 0.170) but a significant interaction of *Age* × *SRC* × *Laterality* (*F*_(2,39)_ = 4.23, *p* = 0.018). *Post hoc* analyses showed that in incongruent SRC trials N2 amplitude was more positive than in congruent SRC trials over midline (incongruent: M = 0.12 μV; SEM = 0.54; congruent: M = −0.10 μV; SEM = 0.50; *p* = 0.023) and right electrodes (incongruent: M = 0.71 μV; SEM = 0.50; congruent: M = 0.50 μV; SEM = 0.46; *p* = 0.036) in elderly adults. However, this difference did not survive Bonferroni correction.

#### P3 Time Window

There was a significant effect for the factors *Laterality* (*F*_(2,39)_ = 9.49, *p* < 0.001) and *Flanker* (*F*_(1,40)_ = 39.76, *p* < 0.001). Furthermore, P3 analysis revealed a significant interaction of *Flanker* × *Age* (*F*_(1,40)_ = 7.64, *p* = 0.009) and *Frontality* × *Laterality* × *Flanker* × *Age* (*F*_(2,39)_ = 3.39, *p* = 0.039). This interaction is based on significant Flanker congruency effects over all electrodes included in the ANOVA in elderly participants (all *p* < 0.001). The difference of Flanker incongruent and congruent trials did not survive Bonferroni correction (all *p* > 0.054) in young adults over all recording sites. In addition, there was a significant effect for the factor *SRC* (*F*_(1,40)_ = 22.90, *p* < 0.001) and a significant interaction of *Laterality* × *Age* × *SRC* (*F*_(2,39)_ = 3.92, *p* = 0.024). Again, elderly exhibited significant SRC congruency effects over left, midline and right electrodes (all *p* < 0.001) while these effects did not survive correction for multiple comparisons in young adults (*p* > 0.063).

In conclusion, data analysis revealed that both conflict types affected RTs, while only the SRC had an effect on error rates. RTs were found to be generally longer in elderly subjects. In addition, the SRC congruency effect on RTs was larger in elderly than young participants. Furthermore, RT analysis showed that RT congruency effects were lager when two sources of conflict were present.

ERP analysis revealed increased P2 amplitudes in response to incongruent Flanker trials, whereas only elderly exhibited an enhanced P2 amplitude in the incongruent SRC condition over right electrodes. Furthermore, the N2 amplitude was more pronounced in the incongruent Flanker condition. Elderly participants elicited an attenuated N2 amplitude in comparison to younger adults. The P3 amplitude in elderly subjects was reduced in response to both conflict types over parietal and centroparietal electrode sides while these effects did not survive Bonferroni correction in young participants. The overall P3 amplitude was also reduced in elderly. There were age-related differences of the ERP scalp topography: the P2 and N2 component peaked over frontal electrodes in young and over frontocentral electrodes in elderly individuals. In addition, the N2 was most pronounced over left electrodes in elderly. In younger subjects the P3 amplitude was most pronounced over parietal recording sites, while elderly exhibited centroparietal P3 peak.

## Discussion

In the present study, we introduced a combined Flanker and SRC conflict task to investigate how aging affects different processing stages of conflict processing and whether these effects are specific for different conflict types. The present results corroborate data from previous studies demonstrating the SRC task to be particularly vulnerable to aging effects. Analysis of RT data yielded an age-related increase of the SRC congruency effect while the Flanker congruency effect did not significantly differ between young and elderly adults. ERP data analysis furthermore demonstrated SRC specific differences between both age groups in the early P2 component. This finding points to age-related differences in early processing stages related to stimulus evaluation that might account for SRC specific deficits in advanced age. Elderly furthermore exhibited an increased modulation of the P3 amplitude in response to incongruent conflict trials. However, this age-related effect was found for both conflict types.

The P2 amplitude was increased in Flanker incongruent trials in comparison to Flanker congruent trials in both groups, while only elderly elicited an amplitude enhancement in response to the incongruent SRC task condition over right electrodes. The sensitivity of the P2 amplitude to conflict processing was also reported by Gajewski et al. (2008) who found that the P2 amplitude is increased in incompatible compared to compatible trials in a response-cueing task in healthy younger adults. Similar data were reported by West et al. (2004) using a numerical conflict task. However, in the present study we could additionally show that the conflict induced modulation of the P2 significantly differed between the two conflict types and age groups. Several studies reported that the P2 amplitude is associated with stimulus evaluation and motivational salience (Luck and Hillyard, 1994; Potts, 2004; Gajewski et al., 2008). In these studies, the P2 amplitude was shown to be enhanced when a specific target feature is task relevant. Another study introducing an oddball task (Kim et al., 2008) demonstrated that the P2 amplitude is increased when the discriminability of the relevant and irrelevant stimulus feature is low. Thus, in the present study the P2 amplitude enhancement in incongruent trials possibly reflects stronger recruitment of neural resources when distinguishing between relevant and irrelevant information. The age-related difference of SRC induced P2 amplitude modulation suggests that this classification process seems to be particularly stressing for the group of elderly subjects while younger adults obviously do not rely on additional resources for SRC related stimulus evaluation. Similar results were reported by Riis et al. (2009). The authors demonstrated that the P2 amplitude is elevated in response to novel stimuli and thus argued that the P2 reflects cognitive processes related to the mismatch of deviant stimuli with a standard stimulus set. Importantly, this effect is particularly pronounced in elderly, indicating that the matching process is possibly enhanced in order to compensate for other age-related alterations. Several studies demonstrated that the processing of distractor stimuli significantly contributes to the decline of conflict processing in elderly, while the processing of relevant stimuli is relatively spared from detrimental aging effects (Gazzaley et al., 2005; de Fockert et al., 2009). The correct identification of the irrelevant stimulus is especially difficult in the SRC task, because relevant and irrelevant features are confounded in the same stimulus (Proctor et al., 2005). Thus, elderly possibly enhance evaluation processes by engaging compensatory cognitive resources to promote the correct discrimination of relevant and irrelevant information input. The enhancement of the P2 amplitude in response to increased attention that was demonstrated in several studies (Daffner et al., 2015) might also indicate that elderly process irrelevant SRC features more intensely. This would imply that higher P2 amplitudes are associated with stronger interference and thus poorer task performance. Indeed, elderly adults show increased SRC congruency effects with regard to RT. However, the study by Daffner et al. (2015) demonstrated that the P2 amplitude positively correlates with better task performance and scores of neuropsychological tests of executive control, suggesting a beneficial or compensatory effect of amplitude enhancement.

The present findings are in line with the fMRI data obtained with same task design and participants (Korsch et al., 2014). fMRI analysis yielded distinctive activation patterns in response to SRC incongruency in young and elderly individuals indicating age-related differences of SRC processing. The incongruent SRC condition was associated with signal increase in the inferior parietal lobule and inferior frontal gyrus of the left hemisphere in elderly participants only. These regions are known to be involved in spatial attention (Culham et al., 2001) and inhibitory and evaluative processes (Downar et al., 2002; Chevrier et al., 2007). Thus, age-related differences of P2 amplitude modulation are possibly related to activation differences in IFG and IPL and reflect corresponding cognitive processes. The frontal topography of the P2 suggests that age-specific differences of the SRC modulation are mediated by regions of the prefrontal cortex. A study by Verleger et al. (1994) revealed that patients with frontal lesions of the left hemisphere showed a marked increase of an early positive component peaking at 150 ms (P150) that was not detectable in patients with lesions of the right frontal cortex or posterior brain regions. Studies investigating neural generators of the P2 in healthy participants are sparse. Potts (2004) demonstrated that the IFG is involved in the neural generation of the P2 in a visual oddball task. These studies point to the fact that the SRC induced P2 modulation in elderly might also be associated with activation of the IFG as observed in the fMRI study. Taken together, early evaluative mechanisms indicated by the P2 component are enhanced in elderly to possibly trigger the correct identification of relevant and irrelevant input and to promote the processing of the mismatch of spatial and color information in incongruent SRC trials. This compensatory mechanism seems to be mediated by the IFG.

In various ERP studies on conflict tasks the incongruent trials exhibited higher N2 amplitudes than congruent trials (for review, see Folstein and Van Petten, 2008) and were associated with early inhibitory control and monitoring processes. Further evidence for this hypothesis is also provided by studies using a source analysis approach (Bekker et al., 2005; Wascher et al., 2011) that demonstrated that the neural generators of the N2 are located in the medial prefrontal cortex, a brain area associated with conflict monitoring, conflict detection, and response selection (Carter et al., 1998; Kiehl et al., 2000; Van Veen and Carter, 2002; Liu et al., 2004). Here, we also found an increase of the N2 amplitude in response to incongruent Flanker stimuli, however, there was no modulation of the N2 component by the SRC task in both groups. Similar data were obtained in a previous study in our lab (Frühholz et al., 2011). Thus, the monitoring and response selection mechanisms indexed by the N2 component seem to be specifically related to the Flanker conflict. This finding corroborates reports that argue for independent neural mechanisms for the processing of different conflict types (Egner et al., 2007; Nee et al., 2007). The present data additionally show that the N2 amplitude is generally more pronounced in younger adults. An age-related attenuation of the N2 amplitude has already been reported in previous studies (Czigler et al., 1996; Amenedo and Diaz, 1998; Bertoli and Probst, 2005; Wild-Wall et al., 2008; Wascher et al., 2011; Lucci et al., 2013; Hsieh and Lin, 2014). Wild-Wall et al. (2008) argued that the more pronounced N2 component in younger subjects possibly reflects a stronger experience of response conflict. They concluded that elderly reduce interference by narrowing the focus of attention to the central target stimulus. This age-related processing strategy change was also observed by Hsieh and Fang (2012).

Furthermore, Cespón et al. (2013) demonstrated age-related differences of the N2pc in a Simon-like task. Interestingly, ERP and behavioral data indicate increased interference effects in young subjects in trials with mismatching color information (target) and arrow direction (distractor). The data furthermore suggested that the lack of interference in this condition in elderly participants is based on an age-related delay of of the processing of the semantic meaning of the distractor (arrow direction) leading to a reduction of the conflict between target and distractor inforamtion. The authors argued that the differences of the N2pc reflect the increased experience of interference in young adults. Similar processes possibly account for the age-related N2 amplitude differences in the present study. However, it must be noted that the behavioral data of the present study rather point to an increase of experienced conflict with regard to the SRC task (direction of arrow) in elderly. Thus, further investigation is needed to clarify the cognitive processes underlying the age-related amplitude differences of the N2 component.

In addition, we found that distinct scalp distributions with regard to N2 amplitude in young and elderly subjects. Young adults elicited peak amplitudes in the N2 time frame over frontal electrode positions while in elderly participants the N2 was most pronounced over frontocentral electrodes.

In accordance with previous studies (van der Lubbe and Verleger, 2002; Wild-Wall et al., 2008) we found age-related differences of the conflict-induced P3 amplitude modulation. Both, the incongruent SRC and the Flanker conflict task conditions were associated with a significantly reduced P3 amplitude extending over parietal and parietocentral recording sites in elderly subjects. In contrast, young adults did not exhibit significant conflict induced amplitude changes over these recording sites. Since the P3 amplitude has been shown to reflect the amount of attentional resources (Polich and Heine, 1996), the decreased P3 amplitude in incongruent trials might indicate that elderly individuals consume more cognitive resources due to higher task demands. A stronger negativity classified as N450 with a broad distribution over central electrodes in response to incongruent stimuli around 300–600 ms after stimulus onset is also consistently found during Stroop task processing. This negativity was furthermore found to be increased in elderly participants. This data is comparable to the increased amplitude reduction in incongruent trials found in elderly adults in the present task. Several Stroop task studies suggest that stimulus-related conflict resolution is decisive for this negative deflection. Mager et al. (2007) thus concluded that elderly use more cognitive resources to resolve conflicting information. This interpretation might also apply for the present data. However, it is still under debate whether the N450 and the P3 represent corresponding or dissociated components. Importantly, according to the present data, the cognitive operations associated with this processing stage seem to be affected equally in different conflict contexts. So, Falkenstein et al. (2002) found that aging effects on the P3 component are less specific regarding task context than the effects on earlier components such as the N2.

The data of the fMRI data of the same task design reported in Korsch et al. (2014) revealed conflict specific activation patterns and aging effects. Extending this finding, the present data suggests that there also seem to be more general effects of aging on interference control as indicated by the modulations of the P3 component. Since neural generators of the P3 have been localized in the temporoparietal cortex this area possibly mediates the P3 amplitude effects detect in the present data.

Taken together, we found that the aging effect on conflict processing seems to be specific for conflict type and the time window of information processing. We furthermore confirmed previous studies that demonstrated that the processing of the SRC task is particularly affected by age-related processes. This was shown by an increased behavioral SRC congruency effect in elderly. The age-specific P2 amplitude increase in incongruent SRC trials suggests that alterations of early processes of stimulus evaluation might be responsible for the SRC-related processing deficit in elderly. In contrast, later stages of information processing associated with the P3 component were found to be altered for both conflict types in elderly participants. This data, in addition to the findings of our previous study (Korsch et al., 2014), suggest that the differential effects of aging on interference control is particularly associated with alterations of early information processing stages associated with stimulus evaluation.

## Author Contributions

All authors listed, have made substantial, direct and intellectual contribution to the work, and approved it for publication.

## Conflict of Interest Statement

The authors declare that the research was conducted in the absence of any commercial or financial relationships that could be construed as a potential conflict of interest.
